# IMPLEMENTATION SCIENCE FOR THE SCALE-UP, SPREAD, AND SUSTAINABILITY OF ASSISTIVE TECHNOLOGIES FOR HEALTHY AGING

**DOI:** 10.1093/geroni/igac059.567

**Published:** 2022-12-20

**Authors:** Shannon Freeman, Simon Carroll

## Abstract

In this symposium we present papers from four key stakeholders on a Michael Smith Foundation for Health Research (MSFHR) Implementation Science Team (IST) that is investigating the application of an emerging approach, the non-adoption, abandonment, scale-up, spread, and sustainability (NASSS) framework (Greenhalgh et al., 2017, 2018), to help predict and evaluate the success of technology-supported health and social care programs in British Columbia (BC). Specifically, our team’s intention is to apply the NASSS framework to answer the following research questions: 1) Can the NASSS framework be used to enhance the effectiveness and impact of innovative ATs through improving the equitable scale-up, spread, and sustainability of these technologies for older adults? 2) What are the most important factors in the technology development and implementation process that contribute to equitable scale-up, spread, and sustainability of ATs for older adults? And 3) How can we improve transdisciplinary and intersectoral collaborations to enhance and improve the equitable implementation of ATs for older adults? Based on discussions from team, advisory, and expert panel meetings, a Rapid Realist Review (RRR) of the literature, and an analysis of data collected to date, the speakers will respond to these questions.

